# Predicting subjective sleepiness during auditory cognitive testing using voice signaling analysis

**DOI:** 10.1186/s41606-025-00141-y

**Published:** 2025-07-01

**Authors:** Tue T. Te, Mary Regina Boland, Sara Ghadimi, Joseph M. Dzierzewski, Cathy Alessi, Jennifer L. Martin, Sarah Kremen, Alex A. T. Bui, Arash Naeim, Constance H. Fung

**Affiliations:** 1 Department of Medicine, David Geffen School of Medicine at University of California, 10833 Le Conte Ave, Los Angeles, CA 90095 USA; 2 Geriatric, Research, Education and Clinical Center, VA Greater Los Angeles Healthcare System, 16111 Plummer Street (11E), North Hills, Los Angeles, CA 91343 USA; 3 Department of Mathematics, Herbert W. Boyer School of Natural Sciences, Mathematics, and Computing, Alex G McKenna School of Business, Economics and Government, Saint Vincent College, 300 Fraser Purchase Rd, Latrobe, PA 15650 USA; 4 National Sleep Foundation, 2001 Massachusetts Ave NW, Washington, DC 20036 USA; 5 Department of Neurology, Cedars-Sinai Medical Center, 127 S. San Vicente Blvd, A6600, Los Angeles, CA 90048 USA; 6 Medical & Imaging Informatics Group, Department of Radiological Sciences, David Geffen School of Medicine at University of California, 924 Westwood Blvd, Suite 420, Los Angeles, CA 90024 USA; 7 Santa Monica, USA

## Abstract

**Background:**

To determine whether objective markers of sleepiness can be collected passively using voice data to detect sleepiness in individuals undergoing testing in situations where sleepiness is not the focal point of assessment. We assessed verbal reaction time (VRT) as a vocalic marker of subjective sleepiness in middle aged and older adults with history of insomnia and benzodiazepine-receptor-agonist (BZRA) use.

**Methods:**

Adults aged ≥55 without a diagnosis of dementia were recruited from a BZRA deprescribing clinical trial and enrolled in the present study that tested the feasibility of cognitive testing using out-of-office, self-directed mobile apps. Participants’ working/episodic memory were assessed through recorded verbal responses to Verbal Paired Associates (VPA) tests, and ecological momentary assessments (EMA) of self-reported sleepiness (1[not at all] to 4[more prominent]). Using a generalized additive model, we examined the association between VRT during VPA testing and self-reported sleepiness, adjusting for demographic, test parameters, caffeine intake, cognition, mood, and BZRA-use (*p*≤0.05 was considered significant). A stratified k-fold cross-validation/random forest (SKCV/RF) was performed to classify sleepiness levels, adjusting for other variables.

**Results:**

We analyzed 1,513 observations from 16 patients. VRT was operationalized as the time duration between recording start time and first speech epoch. Longer VRTs were positively associated with greater EMA sleepiness (*p*≤0.05). The SKCV/RF model yielded a mean F1-score of 0.80 ± 0.08 across folds.

**Conclusions:**

Longer VRTs correlated with greater self-reported sleepiness, indicating that voice data can be used as a marker of sleepiness in patients undergoing cognitive testing in out-of-office settings.

**Supplementary Information:**

The online version contains supplementary material available at 10.1186/s41606-025-00141-y.

## Brief summary

### Current knowledge

Excessive sleepiness is responsible for motor vehicle crashes and other deleterious health effects and can be caused by poor sleep health, sleep deprivation, sleep disorders, drugs, and other conditions. We assessed verbal reaction time (VRT) as a vocalic marker of sleepiness in participants undergoing testing in out-of-office situations where sleepiness is not the focal point of assessment.

### Study impact

Longer VRTs were positively associated with greater self-reported sleepiness (*p*≤0.05). Our prediction model demonstrates the potential of an audio-based approach for real-world sleepiness and cognitive evaluation and monitoring in aging populations.

## Introduction

Excessive sleepiness is responsible for motor vehicle crashes and other deleterious health effects and can be caused by poor sleep health, sleep deprivation, sleep disorders, drugs, and other conditions (Mak et al. 2024). Numerous medications induce sleepiness including benzodiazepine receptor agonists (BZRAs), which are commonly prescribed for insomnia (Mak et al. 2024). Use of BZRAs can also lead to daytime sleepiness and deficits across cognitive domains (Mak et al. 2024; Capiau et al. 2023) and has been associated with increased risk of falls (Glass et al. 2005), especially in older adults. Sleepiness is associated with deficits in decision-making and impairments in cognitive domains including processing speed, working memory, and episodic memory (Kakde et al. 2017). Accurate markers of sleepiness have the potential to be used for identifying individuals at risk of the sequalae of excessive sleepiness (e.g., errors, crashes). Another application is to assess sleepiness passively during medical visits or diagnostic testing such as during cognitive batteries. Excessive sleepiness during cognitive testing, for example, may be a sign of an underlying condition or drug use that could be a reversible or treatable cause of cognitive impairment.

Sleepiness has been measured in a variety of ways, most commonly through (subjective) self-report. The Karolinska Sleepiness Scale assesses sleepiness in the last 10 minutes, conceptualizing sleepiness as a consequence of fluctuation in daily vigilance (state-related) (Shahid et al. 2011). Notably, subjective reports may not always accurately reflect actual sleepiness levels (e.g., in cases where individuals may be penalized for excessive sleepiness such as commercial drivers) (Curcio et al. 2001; Johns 1991). Sleepiness has also been measured objectively as the propensity of an individual to fall asleep (e.g., Multiple Sleep Latency Test) (Johns 2000). Physiological measures have also been used, including pupil diameter variations and electroencephalographic (EEG) parameters (e.g., alpha attenuation index) (Montefusco-Siegmund et al. 2022). In addition, decrements in cognitive and psychomotor tests have also been used to measure sleepiness, including attentional, tapping, and acoustic reaction time tasks. These tests are typically administered in experimental, controlled settings as a discrete test. Measures of sleepiness that can be collected passively and adjunctively are needed.

Voice analysis as a method of detecting sleepiness is an emerging area that offers an alternative to the types of measures described above (Huckvale et al. 2020; Thoret et al. 2024). Initial studies focused on identifying subtle changes in speech patterns associated with cognitive decline. These include changes in speech rate, pauses, pitch variability, word-finding difficulties, and others (Drygajlo et al. 2011; König et al. 2015; Chandler et al. 2023). Researchers have also leveraged advancements in machine learning and signal processing techniques to create algorithms that can analyze these speech characteristics and detect levels of sleepiness (Cheng et al. 2019; Kakde et al. 2017; Inkeaw et al. 2022). Vocal biomarkers, for example, were recently examined in young and middle-aged women who underwent a sleep deprivation protocol, which enabled the researchers to accurately detect sleep deprivation based on machine learning analysis of vocal recordings (Thoret et al. 2024).

The goal of this study was to explore the use of vocalic markers as a measure of subjective sleepiness in middle aged and older adults with insomnia and BZRA use during cognitive testing. We first aimed to measure reaction time indirectly using voice data (verbal reaction time [VRT]). We then sought to explore the link between VRT and self-reported sleepiness. Finally, we aimed to develop a prediction model using ordinal regression to investigate the relationship between VRT and self-reported sleepiness. We hypothesized that longer VRT would be associated with more self-reported sleepiness.

## Material and methods

### Overview

This study tested the feasibility of using smartphones and smartwatches to assess cognition and collect ecological momentary assessments (EMA) in the natural environment (out-of-clinic) of individuals with insomnia and a history of BZRA use. Participants used study-provided iPhone SE smartphones (Apple, Cupertino, California, USA) and Apple Watch 5 or 6 (Apple, Cupertino, California, USA) or their own devices to participate in a verbal cognitive test protocol (Verbal Paired Associates, Digit Span) and complete EMAs over a 28-day period. Participants met with the research team after the 28-day period during a feedback session and provided information about their testing experience. The study was approved by University of California, Los Angeles (UCLA) Institutional Review Board (IRB #20–001519) and all participants provided written consent prior to the study. Data were collected and stored using UCLA’s Research Electronic Data Capture (REDCap) database. Additional details are presented in the Online Supplement.

### Study population

Participants were recruited to the current study from May 2021 to March 2023 from an on-going parent clinical trial (SWITCH, clinicaltrials.gov NCT03687086) conducted at UCLA. The parent trial tested two programs (both using 8 weekly sessions of cognitive behavioral therapy for insomnia [CBT-I] combined with BZRA tapering) to reduce the use of BZRAs and improve insomnia severity in adults aged 55 and older. Briefly, SWITCH enrolled adults aged >55 with current or past use of BZRA medications (e.g., lorazepam, alprazolam, temazepam, clonazepam, zolpidem) for insomnia occurring ≥2 nights per week for at least 3 months for insomnia, and who were able to attend weekly sessions over 9 weeks. Key exclusion criteria included: high risk for complications during hypnotic tapering (e.g., seizure disorder, high baseline dose >8 mg diazepam-equivalent), cognitive impairment (MMSE < 24), unstable medical or psychiatric conditions, polydrug use, active substance abuse, and untreated moderate (accompanied by excessive daytime sleepiness) or severe sleep apnea (Fung et al. 2024).

Inclusion criteria for the BRAIN-e study were English speakers with no cognitive impairment (e.g., Mini-Mental State Examination [MMSE] ≥24).

### Verbal cognitive testing protocol

EMAs were administered daily over a 28-day period. Verbal cognitive testing, including audio recording for Verbal Paired Associates (VPA) and Digit Span tests, was scheduled weekly – Days 1, 8, 15, and 22 of the 4-week study period. Participants were instructed to initiate recordings on these designated days via a unique link to the *Neurovocalix* website (Cambridge Cognition, Cambridge, Massachusetts, USA) (Taptiklis et al. 2017), accessed from the app on their smartphones.

#### Verbal paired associates

The VPA test aimed to assess participants’ associative and episodic memory and consisted of two parts: 1) total recall errors, and 2) delayed recall errors. In the Verbal Paired Associated initial part (VPA-I), participants were audibly presented with eight word pairs and asked to recall which target word best matched each cue word, with up to three attempts allowed for errors. The VPA-I score was calculated as the total number of errors from all attempts ranging from 0 (best) to 24 (worst). For the Verbal Paired Associated delayed recall (VPA-DR), participants recalled the pairs without hearing them again, with a single attempt each week and scores ranging from 0 (best) to 8 (worst).

#### Test difficulty scores

Difficulty scores for Verbal Paired Associate tests were provided by the cognitive testing company and were derived from a logistic mixed effects model considering semantic relatedness measured using cosine similarity from a Global Vectors for Word Representation [GloVe] semantic model, word frequency, and word concreteness (Pennington et al. 2014).

### Ecological Momentary Assessments (EMA)

Self-reported data were collected about the participant’s location, sleepiness, cognitive abilities, mood, alcohol and caffeine intake, and tobacco use. Subjective sleepiness, cognitive abilities, mood, and levels were assessed based on their current state within the past two hours prior to responding. The response options for sleepiness (“In the past 2 hours, how sleepy or drowsy have you felt?”) were (1) not at all, (2) mild, (3) noticeable, or (4) more prominent; and mood (“Your general mood is currently…?”) variables had the following response options: (1) very good, (2) good, (3) fair, or (4) poor. Our measure of subjective sleepiness was based on self-report and was not assessed using standardized or validated tools such as the Epworth Sleepiness Scale or the Karolinska Sleepiness Scale.

#### Other measures

Demographics including data of birth, sex, educational level (number of years), race, and ethnicity were collected as part of the parent trial, and the age of participants at the time of joining the current study was calculated. Whether the participant was taking a BZRA (yes/no) was determined based on the medication log responses from the parent trial using the medication log most proximal to the BRAIN-e enrollment date.

### Data analyses

Additional details are presented in the Online Supplement.

#### Descriptive analyses

Sample characteristics were examined by descriptive analysis to provide an overview of the participants and their self-reported EMA.

#### Voice signaling processing

##### Speech recognition algorithm

Each audio file from VPA-I and VPA-DR tests, corresponding to a participant’s attempt (i.e. “one observation”), was accessed from the *Neurovocalix* platform; processed and analyzed using package *tuneR* (version 1.4.7) (Ligges et al. 2023), *seewave* (version 2.2.3) (Sueur et al. 2008), and *signal* (version 1.8-1) (Signal developers 2014) in R (version 2023.12.1+402). Sessions that participants self-identified as instances of using written notes to assist with recall (“invalid strategies”) were excluded from the analysis. The left channel of each audio file was normalized (e.g., to a range of [−1,1]) to facilitate uniform analysis regardless of variations in overall signal intensity across recordings. A threshold of 0.3 was empirically set to detect the start of audio signals based on their amplitude ground (Barchiesi and Droppo 2014; Institute of Electrical and Electronics Engineers n.d.).

##### Validation

Out of 2,230 audio samples, 185 (8.3%) were randomly selected for manual validation. The validation process involved verifying whether recordings contained speech or non-speech sounds, further checking the accuracy of start times. Visual and auditory validation were performed using Mel-frequency Cepstral Coefficients (MFCCs) plots in R (*seewave* package) to compare calculated start times with manual inspection.

##### VRT

The start time was defined as the first audio sample where amplitude exceeded the threshold, marking the beginning of speech. VRT was measured from this start time to the first segment of speech. Statistical differences between sexes were assessed using t-test, Mann-Whitney U test, and variance test.

#### Statistical analyses

EMA data collected on the days of the recordings from VPA tests (Days 1, 8, 15, 22) were used. The audio tests flagged instances of self-reported “invalid strategies” and days missing EMA data, which were excluded.

##### Linear regression (LR) analyses

For feature selection, a random forest (RF) was used to assess predictor importance for sleepiness scale. The ranking of features was determined based on an increased node purity value, which was used as the threshold for selecting the most influential predictors. Then, a linear regression (LR) model was fit with all predictors. A forest plot was generated to visualize the associations of these variables with sleepiness (probability values were considered significant with *p*≤0.05). A regression equation specification error test (RESET) (Lee et al. 1993) was performed to evaluate if adding higher-order (nonlinear) terms could improve the model fit.

##### Generalized additive model (GAM) analysis

A GAM is a flexible statistical method designed to handle both linear and nonlinear relationships between a response variable and several predictors by employing smoothing/spline functions (Hastie and Tibshirani 1986). In this analysis, the outcome variable was sleepiness, and the primary predictor of interest was VRT. VRT, age, education, test difficulty, and caffeine intake were modeled using smooth functions to capture their non-linear relationships (Hastie and Tibshirani 1986). Sex, BZRA status, mood, and cognitive scale were treated with linear terms. The *mgcv* package (version 1.8–40) using R (version 4.3.1) was used (Wood 2011). Alpha was set at 0.05 with probability values less than or equal to 0.05 considered statistically significant. Akaike Information Criterion (AIC) values for the linear regression model and GAM were calculated to evaluate and compare their relative goodness of fit (Burnham and Anderson 2002). An ANOVA test was performed to test for significance between the two models.

#### Stratified K-fold cross-validation & random forest (SKCV/RF) prediction model

Given the class imbalance and relatively small dataset, we performed a stratified k-fold cross-validation using the *caret* package (version 6.0–94) (Kuhn 2008) in R (version 4.3.1) for data preparation and cross-validation. In contrast to the GAM model, this new model was built at the patient level (i.e., each patient was considered one *observation*). Here, patients were balanced based on their class distribution. Unique patient identifiers were used to create distinct training and testing subsets, ensuring that each fold used a different set of participants for training and testing, and for a given set, the training and testing sets were mutually exclusive (i.e., a patient could not both be in the training and testing set simultaneously). Four stratified folds were generated based on the sleepiness scale, ensuring that each fold contained a similar distribution of sleepiness levels. For each fold, the training data was balanced by class. If a class had fewer than 180 samples, it was skipped; and if a class had more than 500 samples, it was downsampled to 500. This balancing step ensured no samples from the same time point and same patient would be split between training and testing sets. We performed feature selection using RF on each fold’s training data to identify the most influential predictors. The same increased node purity value was applied across all folds’ feature selection models. Those features consistently ranked as unimportant across all folds were removed (i.e., variables with a value below 20 across all folds were considered less influential and removed). A random forest classifier was then used to predict sleepiness levels based on the independent variables. *Bootstrapping* was performed with 1,000 iterations. The model was trained using the following predictors: VRT, cognition scale, mood scale, age, sex, caffeine intake, benzodiazepine use, education, and test difficulty. The model’s performance was assessed on the holdout folds (testing dataset) using a confusion matrix, accuracy, precision, recall, and F1-score statistic. 95% Confidence intervals were also computed on each fold. The final performance metrics were averaged across the four folds for overall performance.

## Results

Additional details are presented in the Online Supplement.

### Sample characteristics of primary analysis

**Fig. 1:**
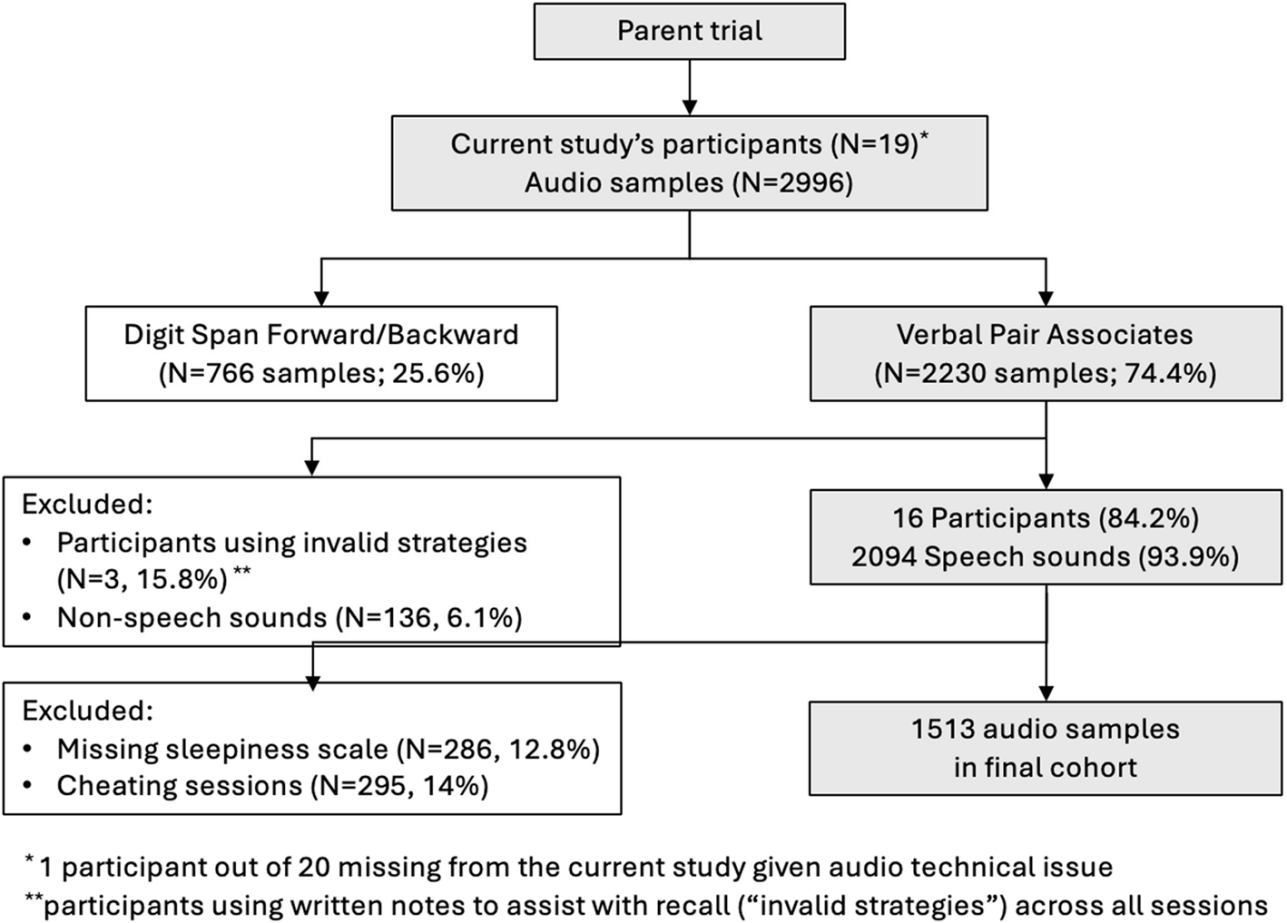
Flow diagram of audio samples for analysis and prediction model subset

Figure 1 illustrates the selection process for our cohort. Of the participants enrolled in the parent trial, 20 were recruited to participate in the BRAIN-e study. Four were excluded – one due to technical issues and three for self-reported use of *invalid strategies* during testing. From 2,996 audio samples, 2,230 (74.4%) were selected for VPA analysis, consisting of 2,094 (93.9%) speech sounds and 136 (6.1%) non-speech sounds. After removing non-speech sounds, *invalid strategies* sessions (*N*=295, 14%), and missing sleepiness data (*N*=286, 12.8%), 1,513 samples remained. The final generalized additive model cohort (*N*=16) had a median age of 65.5 years (interquartile range [IQR] 63.4–70.5), with 68.8% female participants. Most were White (87.4%) and had a median education of 16 years in school (IQR 16–18) (Table 1).

**Table 1:** Characteristics of participants included in speech analysis

Characteristics		*N*=16
Age	Mean (SD)	66.34 (5.1)
	Median	65.5
	IQR	63.4, 70.5
	Range	56.8, 73.6
Sex (%)	Female	11 (68.8%)
	Male	5 (31.2%)
Race (%)	Black or AA	1 (6.3%)
	Hispanic or Latino	1 (6.3%)
	White	14 (87.4%)
Education (years)	Mean (SD)	16.5 (1.9)
	Median	16
	IQR	16, 18
	Range	12, 20

### EMA and other variables

Descriptive analyses of the EMA data from 1,513 sessions reveals notable trends. Table 2 showed the variation in sleepiness levels across 16 participants over 4 or 5 weekly sessions, with levels ranging from 1 (not at all) to 4 (more prominent). Across all sessions, sleepiness was most commonly reported as mild (level 2) (39.5%), 23.7% of sessions indicating no sleepiness (level 1), 19.8% more prominent (level 4), 17.1% noticeable (level 3). Sleepiness levels were generally mild (2), with many participants (9 out of 16) reporting a level of mild (2) or not at all (1) during week 1. However, there was considerable variability over time. Some participants showed consistency in their sleepiness levels, such as Participant 9, who maintained a level of 1 (“not at all”) for the first three weeks, while others exhibited fluctuations, like Participant 8, whose sleepiness increased from mild (2) to more prominent (4) by Week 4. Several participants had missing data due to non-recorded sleepiness level during the sessions, technical issues, or ‘invalid strategies’ for multiple weeks, which limited the ability to assess trends across the full 4 or 5-week period.

**Table 2:** Distribution of sleepiness level across participant over all sessions

**Participant**	**Sleepiness level**				
	**Session Week 1**	**Session Week 2**	**Session Week 3**	**Session Week 4**	**Session Week 5**
1	2	2	2	3	N/A
2	2	N/A	N/A	N/A	N/A
3	2	2	2	3	3
4	2	2	2	N/A	N/A
5	4	4	2	1	N/A
6	N/A	3	N/A	N/A	N/A
7	N/A	3	N/A	N/A	N/A
8	2	4	4	4	N/A
9	1	1	1	2	N/A
10	1	4	4	4	N/A
11	1	2	1	1	N/A
12	2	2	3	2	4
13	1	3	3	2	N/A
14	2	N/A	N/A	N/A	N/A
15	1	N/A	1	N/A	N/A
16	N/A	N/A	3	N/A	N/A

*NA* Not applicable

Descriptive statistics summarizing other EMA questions are described in the online supplement (Table S1).

### Voice signaling processing and validation

Raw audio data from 2,996 files were extracted from the Neurovocalix platform:

1. STEP 1 (initial algorithm to separate speech and non-speech): Of the 2,996 files, 2,230 samples from the VPA and VPA-DR tests were selected for analysis. An empirical threshold of 0.3 was used to classify audio as speech or non-speech sounds. This threshold identified 2,080 samples (93.2%) as speech and 150 samples (6.7%) as non-speech.
2. STEP 2 (manual validation of a random subsample): To ensure the accuracy of this automated classification, we randomly selected a subset of 185 samples, which represented 8.3% of the total dataset, and used the model to predict speech and non-speech for this subset. Speech was predicted in 35 samples, and non-speech was predicted in 150 samples. Among the 35 predicted speech samples, our manual validation found that all 35 had speech (i.e., “true speech”), which yields a positive predictive value (PPV) of 100% (the samples initially labeled as speech by the algorithm were indeed real speech). Among the 150 predicted non-speech, our manual validation found that 136 had no speech (i.e., “true non-speech”), yielding a negative predictive value (NPV) of 91% and demonstrates that there were 14 samples that were initially misclassified as non-speech, yielding an overall accuracy of 92.5% in correctly identifying speech vs. non-speech. Table S2 summarizes the results of manual annotation: there were 14 false negatives, 0 false positives, 35 true positives, and 136 true negatives. We were more interested in predicting speech because we were modeling speech and those recordings where the individual spoke were of most interest and therefore were included in our model whereas those who did not speak or were predicted to not speak by the algorithm were excluded.
3. STEP 3 (manual correction of total sample): We manually pulled the 14 mislabeled samples that were originally labeled as non-speech but were true speech, and we put these 14 samples into the true speech dataset such that the final dataset had a total of 2,230 samples. Of these, 2,094 samples (93.9%; 2,080+14 samples) were speech, and 136 samples (6.1%; 150-14 samples) were non-speech. From STEP 1 to STEP 3, the percentage of non-speech samples decreased from 6.7% to 6.1% due to the 14 samples that we manually corrected their proper classification as speech sounds.

For the 1,513 speech sounds analyzed, the median VRT was 1.68 seconds (IQR 0.89–2.91), with a mean of 2.19 ± 1.80 seconds. Reaction times were slightly longer in males, with a median of 2.22 seconds (IQR 1.82) compared to 2.18 seconds (IQR 1.79) in females. Statistical tests indicated no significant differences between the sexes with mean (*p*=0.72), median (*p*=0.67), or variances (*p*=0.75). The accuracy of voice signal processing to assess VRT to detect true speech is based on the original performance (before the manual correction step) to ensure it reflects the true performance of the model (92.5%).

### Statistical analysis

**Fig. 2:**
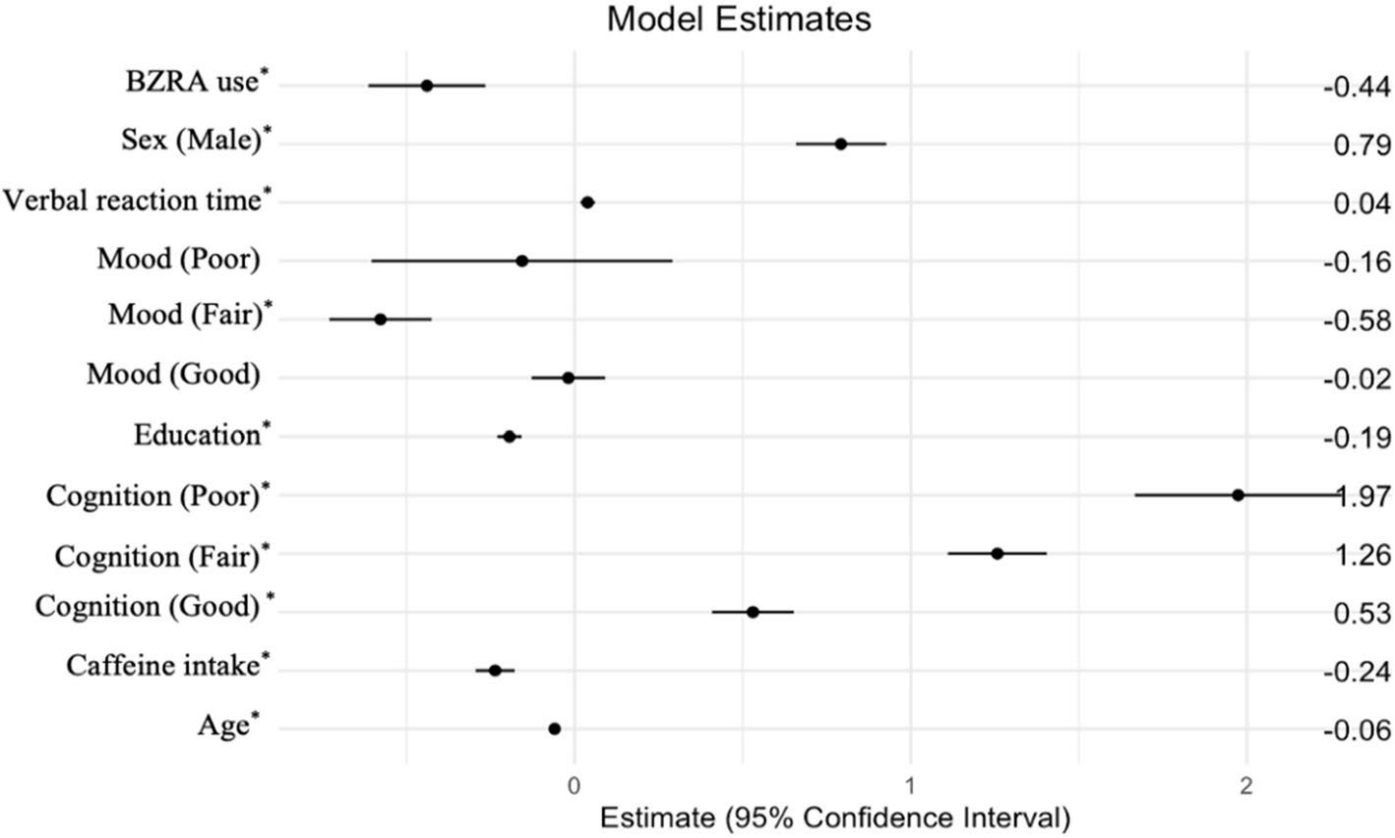
Forest plot for linear regression model

First, random forest analysis was used to identify key predictors of sleepiness by measuring the importance of each predictor variable. Variables with an increased node purity value below 50 were deemed less influential; therefore, *pass/fail* and *alcohol use* were removed. *Race* was omitted to avoid potential issues related to imbalance effects. Second, the linear regression model was fitted with the remaining predictors that showed VRT, cognition, age, caffeine, education, mood (fair), sex, BZRAs use were significant predictors of sleepiness (*p*≤0.05). The forest plot (Fig. 2) showed that lower cognitive performance (higher levels on cognitive scale) and male sex were associated with increased sleepiness. Older age, increased caffeine use, higher education, the use of BZRA were associated with reduced sleepiness. However, this model only explained approximately 58.6% of the variance in sleepiness. The *p*≤0.05 from the RESET test indicated strong evidence against the null hypothesis of the linear model being adequate, which suggested there is a significant amount of nonlinearity present in the data that the linear model fails to capture.

**Fig. 3:**
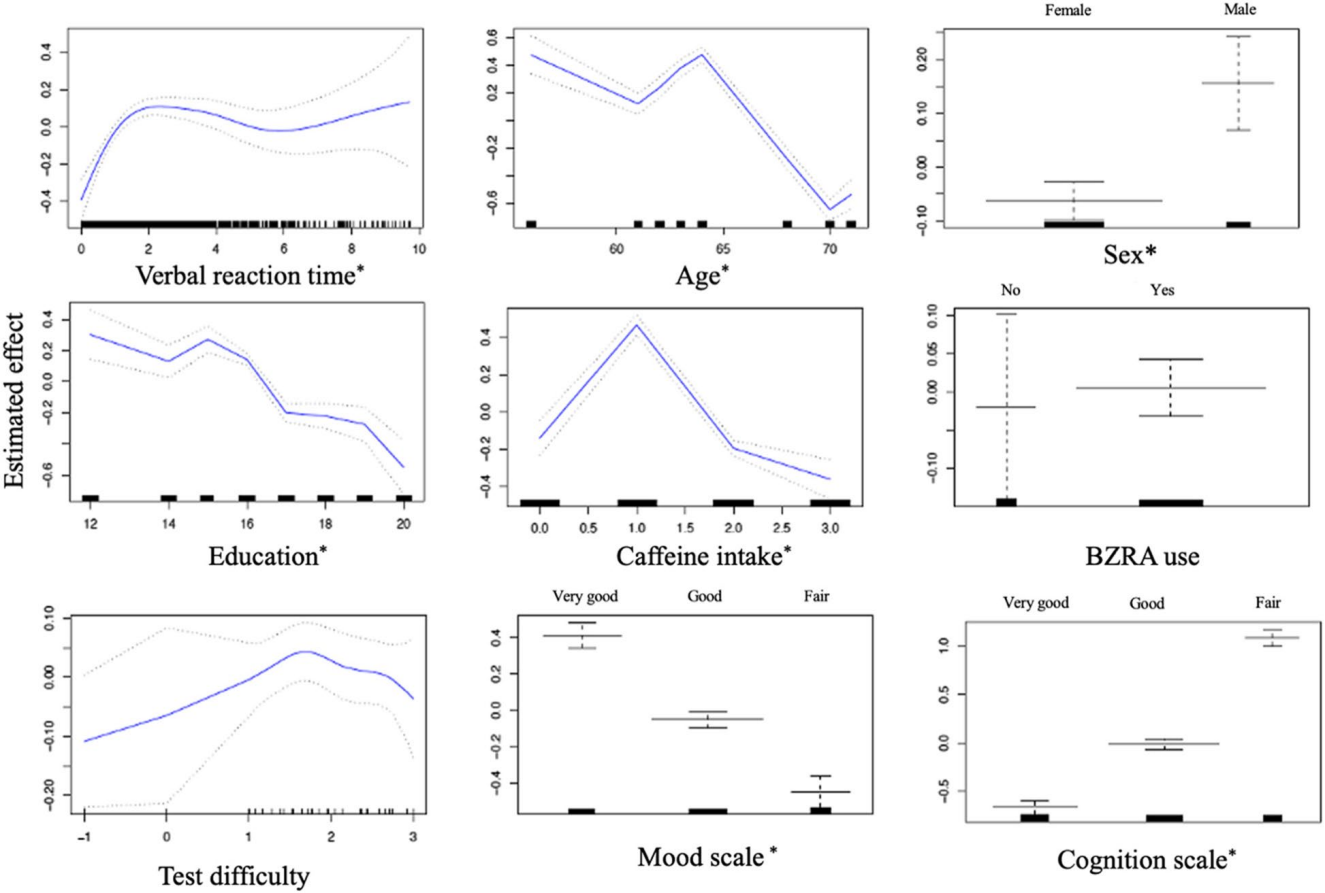
Generalized additive model (GAM) result. BZRA: Benzodiazepine Receptor Agonist. ^*^probability values were considered significant with *p*≤0.05

To address this nonlinearity, we thus constructed a generalized additive model. The GAM analysis, used as the final model to explore predictors of sleepiness, incorporated smooth functions for continuous variables (VRT, age, education, caffeine intake, and difficulty) and parametric terms for sex, cognitive scale, and mood. In Fig. 3, the analysis revealed that longer VRT durations, male sex, and lower cognitive levels were associated with increased sleepiness, while older age (peaking at 64 years), higher education (peaking at 15 years), higher caffeine intake, and good/fair mood levels were linked to decreased sleepiness (*p*≤0.05). BZRA use and test difficulty were not significant predictors (*p*>0.05). With an Akaike Information Criterion of 3078.57 compared to 3478.35 for the linear regression mode, the generalized additive model more effectively explains the variability in sleepiness than linear regression model it also captured elements of nonlinearity. The ANOVA test indicates significant differences (*p*≤0.05), further supporting the superiority of the GAM over the linear model.

### Stratified K-fold cross-validation and random forest (SKCV/RF) prediction model

To prevent data leakage, we first performed SFCV to define four mutually exclusive training and testing folds at the patient level. Feature selection was conducted only on the training set within each fold using a RF model to identify the most influential predictors. To ensure fairness and consistency across folds, we applied the same cutoff for importance based on the increased node purity value for each fold. Variables with a value below 20 were considered less influential and candidates for removal. *Alcohol use* and *pass/fail* status had importance values consistently below 20 across all four folds. *BZRA status* and *test difficulty* fell below this threshold in some folds but not all. Consequently, we removed *alcohol use* and *pass/fail* status from the final model as they were uniformly deemed less important across all folds. The model was trained using the following predictors: VRT, cognition scale, mood scale, age, sex, caffeine intake, education, BZRA status, and test difficulty.

In Table 3, the results from the SKCV/RF demonstrate varied performance across the folds, reflecting the model’s ability to predict sleepiness levels with different levels of accuracy and consistency. Accuracy ranged from 0.56–0.76 with mean of 0.64 ± 0.09, with a single fold (Fold 1) showing the highest accuracy (0.76, 95% CI: 0.69–0.83), suggesting that the model performed well in predicting sleepiness for this subset of data. However, performance declined in the subsequent folds, with ensuing folds showing lower accuracy (e.g., Folds 2 & 3; 0.58 [95% CI: 0.50–0.66] and 0.56 [95% CI: 0.48–0.64], respectively), indicating that the model struggled with these splits. Precision across the folds was relatively good, ranging from 0.68 to 0.76 with mean of 0.73 ± 0.04, indicating a strong ability to correctly classify positive predictions of sleepiness, with the first fold achieving the highest precision (0.76 [95% CI: 0.69–0.83]). Recall showed stronger consistency with mean of 0.84 ± 0.11, peaking at 1.00 in Fold 1, where the model perfectly identified all instances of sleepiness, but decreasing in subsequent folds (ranging from 0.75 to 0.8). This result suggests that while the model was able to capture most true positives, the best values were observed in the first fold. The F1 score, which balances precision and recall, ranged from 0.70–0.90 with mean of 0.80 ± 0.08, with generally strong performance in Folds 1 & 2, with Fold 4 showing the lowest F1 score (0.70 [95% CI: 0.63–0.77]), likely due to the trade-off between precision and recall in that fold. Overall, the model’s performance with bootstrapping showed variation across the different folds, with some folds achieving high precision and recall, while others exhibited more moderate results, suggesting the potential for refinement and further optimization in future.

**Table 3:** Performance metrics from stratified k-fold cross-validation/random forest

**Fold**	**Accuracy (CI 95%)**	**Precision (CI 95%)**	**Recall (CI 95%)**	**F1 (CI 95%)**
1	0.76 (0.69; 0.83)	0.76 (0.69; 0.83)	1.00 (1.00; 1.00)	0.90 (0.85; 0.95)
2	0.58 (0.50; 0.66)	0.70 (0.63; 0.77)	0.8 (0.76; 0.86)	0.80 (0.76; 0.86)
3	0.56 (0.48; 0.64)	0.76 (0.69; 0.83)	0.79 (0.72; 0.86)	0.80 (0.76; 0.86)
4	0.65 (0.57; 0.73)	0.68 (0.61; 0.76)	0.75 (0.68; 0.82)	0.70 (0.63; 0.77)
Mean ± SD	0.64 ± 0.09	0.73 ± 0.04	0.84 ± 0.11	0.80 ± 0.08

## Discussion

We identified voice VRT as a potential vocalic marker of sleepiness in a sample of middle aged and older adults with a history of insomnia and BZRA use undergoing out-of-center self-administered cognitive testing, with our SKCV/RF models in predicting subjective sleepiness levels yielding a mean F1-score of 0.80 ± 0.08 across folds. Our voice signal processing procedures, which we manually validated, yielded high accuracy (92.5%) of classifying audio data as speech (versus non-speech) in this sample of older adults who were undergoing cognitive testing. Our findings highlight the potential of voice signaling processing and the algorithm as a valuable tool in detecting subjective sleepiness using voice data and contribute to the understanding of vocalic markers in assessing cognitive function and sleepiness, particularly in older populations.

This study’s novel approach to assessing subjective sleepiness through VRT derived from voice signal processing during cognitive testing provides information that is unavailable through traditional pen-and-paper cognitive tests. We hypothesized that greater self-reported sleepiness would correlate with longer VRTs during cognitive tasks. Unlike previous studies focusing on pass/fail status or speech features, our study is among the first to objectively measure VRT from audio recordings. Our analysis used a series of analytical methods to confirm that longer VRTs are associated with increased sleepiness after adjusting for multiple factors, supporting earlier research linking sleepiness to impaired cognitive performance and slower reaction times (Jafari et al. 2020). While VRTs were slightly longer for males in our study, this difference was not statistically significant, contrary to some literature which often reports faster reaction times in males (Vamsi et al. 2023).

The GAM analysis was used as the final model. Unlike traditional linear models, GAMs allow for the inclusion of nonlinear relationships between predictors and outcomes. In our complex dataset, the relationship between age and sleepiness was nonlinear. Sleepiness was found to peak in individuals around the age of 64 years, with older adults experiencing a relative decrease in sleepiness levels beyond this age. This pattern may reflect the complex interplay between aging and sleep regulation. As individuals age, they may experience changes in sleep architecture and circadian rhythms that affect sleepiness. The nonlinear relationship here may suggest that there is a certain age threshold (or other point), after which individuals may have more stable or less pronounced sleepiness levels, potentially due to different sleep patterns, reduced work demands, or medical treatments. Higher education was found to be associated with decreased sleepiness, with the effect peaking at 15 years of education. This may then suggest that individuals with more years of formal education might have better sleep hygiene, more structured routines, or fewer factors contributing to chronic sleepiness. Still, it is also possible that education reflects socioeconomic factors or cognitive reserve, which may help individuals manage stressors and sleep disturbances better, leading to lower sleepiness levels. Mood was another key predictor of sleepiness. Individuals in good or fair mood had notably lower sleepiness, while those with poorer mood states may report higher levels of sleepiness. Individuals reporting positive mood states tend to experience less sleepiness, possibly due to the psychological and physiological benefits of positive affect, which can enhance energy and alertness; while poor mood states could exacerbate sleepiness, potentially due to negative effects of emotion, which can interfere with sleep quality and increase feelings of fatigue. On the other hand, heightened sleepiness may also worsen mood, creating a bidirectional relationship. Further investigation into the interplay between mood and sleepiness is warranted to better understand the causal mechanisms and feedback loops that may contribute to these associations.

Our study’s prediction model performed relatively well in predicting sleepiness levels, achieving F1-scores from 70% to 90% across four categories. Compared to Gao et al.’s study that focused on detecting fatigue through speech analysis with added physiological indicators including P300 responses and saliva glucocorticoid levels (94% accuracy) (Gao et al. 2022), our algorithm had lower accuracy ranging from 56–76% with mean of 64% ± 0.09, yet was more practical and less intrusive using voice data. Kaser et al.’s 88.4% accuracy in detecting cognitive impairment further supports the effectiveness of voice-based assessments using speech-to-text transcription and digital-signal processing (Kaser et al. 2024). While Kaser et al. focused on cognitive impairment, their results highlight the potential of voice-based assessments for differentiating cognitive states. Our model’s good F1 score of mean of 80% demonstrates that using SKVC/RF and VRT could offer an alternative and simple method for predicting sleepiness compared to earlier approaches. In addition, our stratified K-fold cross-validation was performed to address possible class imbalance. The SKCV/RF prediction model performs strongest in recall and F1 score, suggesting that the model is particularly effective at identifying true positives (i.e., correctly identifying instances of sleepiness) while minimizing false negatives. A high recall value (0.75[95%CI: 68%−82%]−1.00[95%CI: 100%−100%] across 4-folds) indicates that the model has successfully detected most of the actual cases of sleepiness, which is critical in applications where missing a case of sleepiness could have serious consequences. The F1 score is the harmonic mean of precision (positive predictive value) and recall (sensitivity). This is particularly useful when the class distribution is imbalanced, as it balances the trade-off between precision and recall. A high F1 score (range: 70%[95%CI: 63%−77%]−90%[95%CI: 85%−95%]) can suggest that the model performs well at identifying sleepiness accurately (high precision) and capturing all instances of sleepiness (high recall). This makes the model useful not just for detecting sleepiness, but for ensuring the detection process is both accurate and complete. However, the variation in accuracy and recall across the folds suggests there may be areas for improvement, particularly in ensuring that the model generalizes well to all subsets of the data. These fluctuations could be addressed through techniques such as training in a larger dataset, optimizing data balance, and exploring additional features.

Developing markers of sleepiness during cognitive testing is important because sleepiness can impact cognitive performance, which might confound or lessen confidence in results obtained by cognitive assessments. In the Washington Heights-Inwood Community Aging Project, excessive daytime sleepiness (EDS) in older adults was associated with slower processing speeds, potentially indicating early cognitive decline (Tsapanou et al. 2016), and in another study (Ohayon and Vecchierini 2002), EDS was linked to higher risk of cognitive impairment across multiple domains. Speech-based research (Fagherazzi et al. 2021) leverages the unique characteristics of human voice to serve as acoustic markers (Chandler et al. 2023). Anatomical variation in the vocal tract anatomy, histopathological changes observed with aging, and alterations due to physiological states and illnesses contribute to unique patterns that can be measured (Chandler et al. 2023; Kuhn 2014). Speech-based diagnostics have included general-purpose low-level descriptors, short-term spectrum and energy, or advanced methods like bag-of-audio-word encoding (BoAW) and convolutional neural network (CNN) trained on raw speech waveforms or Mel-spectrograms (Egas-López JV and Gosztolya 2021; Gosztolya 2019).

Our study has several strengths. A comprehensive analytical approach including LR and GAM was able to model (non)linear effects that enhance the model’s accuracy and applicability. Our approach, which objectively measures VRT from audio recordings, provides a novel, non-invasive, cost-effective method, suitable for real-world assessments and remote monitoring. This can be particularly useful when subjective reporting is unreliable or unavailable. The potential benefits of VRT-based sleepiness assessment include improved accuracy, early detection of sleepiness which could be related to sleep-related issues or cognitive issues, and integration into remote or passive monitoring platforms, such as telehealth or mobile apps. Using the validated algorithm we developed in this project, we are actively working toward full automation using validated speech-processing algorithms to enhance scalability. We believe this approach complements traditional self-reports and holds promise for advancing clinical assessment and monitoring of sleepiness in aging populations. As a next step, we hope to conduct a prospective study to further validate VRT as an objective marker of sleepiness and evaluate its predictive utility in real-world and clinical environments.

Despite the promising results, our study has several limitations. The sample size of 1,513 audio samples comes from 16 participants(minimum age 56, maximum age 73) and therefore is relatively small. We attribute the age distribution of our sample to recruitment from the parent trial. Middle-aged and older adults are more likely to undergo cognitive testing for issues such as memory impairment than children and young adults. The parent trial from which participants for this ancillary study were recruited targeted older adults, who are at greater immediate risk for side effect from BZRAs, and middle-aged adults, who are at future risk for side effects from BZRAs. Subjective sleepiness was not assessed using standardized or validated tools such as the Epworth Sleepiness Scale or the Karolinska Sleepiness Scale. The analysis focused solely on VRT without account for other factors like other sleep disorders (e.g., sleep apnea), other medications (e.g., opioids), environmental influences (e.g., in a noisy environment, an individual may speak more slowly or more loudly to ensure they are heard clearly), and normal individual differences (such as personality, cultural communication norms, and so forth) that may influence the VRT. The VRT in this study was estimated during a controlled cognitive task that could differ from natural speech, where an individual’s reaction time and speech characteristics would reflect their unprompted, real-world interactions, typically under less structured or more relaxed conditions. Validating speech patterns in ambient environments is indeed an essential next step to further enhance our understanding of how unique audio characteristics relate to clinical outcomes. The class imbalance in the predicted variables, particularly with respect to the distribution of self-reported sleepiness scores, could present a challenge. While the distribution of sleepiness scores in this dataset does not seem to present significant problems, class imbalance could still affect model performance and generalizability. Specifically, models may struggle to detect sleepiness in underrepresented groups if certain levels of sleepiness are under-sampled. Although stratified k-fold cross-validation can mitigate some of the effects of class imbalance by ensuring that each fold contains a proportionate representation of each class, this approach may not fully address the underlying imbalance. Future work should validate the model on a more balanced dataset, ensuring that all categories are adequately represented. The validation of speech versus non-speech categories was performed manually by a single person that may introduce subjective bias. Although the accuracy rate for the speech analysis to detect true speech and calculate VRT is high (92.5%), there may still be some samples mislabeled as *non-speech* but that are actually *true speech*. Specifically, based on our manual validation, where 14 out of 150 samples predicted as non-speech were true speech, we would expect approximately 13 *true-speech* samples in the full dataset to be incorrectly labeled as non-speech. Reliance on self-reported data also introduces potential bias, and audio quality issues could affect the accuracy of voice signaling and analyses. Although time of day data was collected, it was not included as a covariate in the current analysis. This is an important area for future analysis given that time of day may influence sleepiness levels. Additionally, the cross-sectional design of this study limits causal inferences.

## Conclusions

In conclusion, this study demonstrates the potential of an audio-based approach for real-world sleepiness and cognitive evaluation and monitoring in aging populations. Future research should address limitations of the current study including employing larger samples to improve the robustness and applicability of voice-based assessments for cognitive function and sleepiness. Longitudinal studies are needed to establish the temporal dynamics and causality of these associations.

## Supplementary Information

Supplementary Material 1.

## Data Availability

No datasets were generated or analysed during the current study.

## Abbreviations

VRT: Verbal Reaction Time
BZRA: Benzodiazepine Receptor Agonist
UCLA: University of California, Los Angeles
VPA: Verbal Paired Associates
GAM: Generalized Additive Model
CBT-I: Cognitive Behavior Therapy For Insomnia
MMSE: Mini-Mental State Examination
DGS-F: Digit Span Forward
DGS-B: Digit Span Backward
EMA: Ecological Momentary Assessments
ESS: Epworth Sleepiness Scale
EEG: Electroencephalographic
LR: Linear regression
RF: Random Forest
GloVe: Global Vectors for Word Representation
RESET: Regression Equation Specification Error Test
AIC: Akaike Information Criterion
ETC: Extra Tree Classifier
SKCV/RF: Stratified k-fold cross-validation/random forest
IQR: Interquartile Range
EDS: Excessive Daytime Sleepiness
