# Supplementary material for: Predicting subjective sleepiness during auditory cognitive testing using voice signaling analysis
Source: Sleep Sci Pract. 2025 Jul 1;9(1):19. doi: 10.1186/s41606-025-00141-y (PMC12213935; doi:10.1186/s41606-025-00141-y)
Supplement: Supplementary file 1 — Supplementary Material 1. [file 41606_2025_141_MOESM1_ESM.docx]

**ONLINE SUPPLEMENT**

**Predicting Sleepiness During Auditory Cognitive Testing Using Voice Signaling Analysis**

Tue T. Te, MD, MBMI^1,2^, Mary Regina Boland, MA, MPhil, PhD, FAMIA^3*^, Sara Ghadimi, BS^2^, Joseph M. Dzierzewski, PhD^4^, Cathy Alessi, MD^1,2^, Jennifer Martin, PhD^1,2^, Sarah Kremen, MD^5^, Alex A.T. Bui, PhD^1^, Arash Naeim, MD, PhD^1,2^, Constance H. Fung, MD, MSHS^1,2*^

*Co-senior authors

Corresponding author: Tue Te, MD. Address: 1260 15^th^ Street, Suite 600, Santa Monica, CA 90404, California, USA. Email: TTe@mednet.ucla.edu. Tel: 310-449-0939. Fax: 424-259-7790

Institution where the work was performed: University of California, Los Angeles, CA, USA; VA Greater Los Angeles, Los Angeles, CA, USA

**Supplemental Methods** 2

**Supplemental Results** 3

**Supplemental Discussion** 4

**Table S1:** Distribution of Ecological Momentary Assessments variables across all sessions 6

**Table S2:** Result of the Manual Annotation 7

**Supplemental References** 8

**SUPPLMENTAL METHODS**

**Verbal Paired Associates.** While this study primarily focused on the Verbal Paired Associates, Digit Span tests were administered after the VPA-I test and prior to the VPA-DR, serving as a distractor task to remove items from short-term memory and are integral to calculating the difficulty score used in the current analysis. In the Digit Span-Forward test, participants were presented with a sequence of numbers (e.g., "2-8-3-4") and instructed to repeat them, starting with two digits and gradually increasing up to a maximum sequence length of eight digits, with the test concluding when they failed to correctly recall the sequence in three consecutive attempts at the same length. In the Digit Span-Backwards test, participants were instructed to repeat the numbers in reverse order. These Digit Span tests provide a standardized measure of working memory capacity, which is crucial for determining the difficulty score utilized in our analytical approach.

**Ecological Momentary Assessments (EMA)**

EMA data were collected at the same time across days and on the same days as audio recordings from VPA tests (days 1, 8, 15, and 22). Data were collected about the participant’s location, sleepiness, cognitive abilities, mood, alcohol and caffeine intake, and tobacco use. Sleepiness, cognitive abilities, mood, and levels were assessed based on their current state within the past 2 hours prior to responding. The response options for sleepiness (“In the past 2 hours, how sleepy or drowsy have you felt?”) were (1) not at all, (2) mild, (3) noticeable, and (4) more prominent. Cognitive ability (“Your general thinking abilities are currently…?” and mood (“Your general mood is currently…?”) variables had the following response options: (1) very good, (2) good, (3) fair, and (4) poor. Alcohol and caffeine intake were recorded based on the number of servings consumed that day. Tobacco use reflected whether participants had used tobacco products or not.

**Other Measures**

Participants who indicated during their feedback session that they had used test strategies that would invalidate the cognitive test results (e.g., writing down cues or responses) were asked which sessions they had used these strategies, and were deemed “invalid.”

**SUPPLMENTAL RESULTS**

**EMA and other variables.** Descriptive analyses of the EMA data from 1,513 sessions reveals notable trends. Across all sessions, sleepiness was most commonly reported as mild (level 2) (39.5%), 23.7% of sessions indicating no sleepiness (level 1), 19.8% more prominent (level 4), 17.1% noticeable (level 3). Table S1 shows the variation in sleepiness levels across 16 participants over 4 or 5 weekly sessions, with levels ranging from 1 (not at all) to 4 (more prominent). Sleepiness levels were generally mild (2), with many participants (9 out of 16) reporting a level of mild (2) or not at all (1) during week 1. However, there was considerable variability over time. Some participants showed consistency in their sleepiness levels, such as Participant 9, who maintained a level of 1 ("not at all") for the first three weeks, while others exhibited fluctuations, like Participant 8, whose sleepiness increased from mild (2) to more prominent (4) by Week 4. Several participants had missing data due to non-recorded sleepiness level during the sessions, technical issues, or ‘invalid strategies’ for multiple weeks, which limited the ability to assess trends across the full 4 or 5-week period.

Descriptive statistics summarizing other EMA questions demonstrate that participants generally rated their cognition positively: 34.1% considering their current cognitive abilities as very good, 44.3% as good, although 4.2% as poor. Mood assessments were also predominantly positive: 31.3% very good among all sessions, 45.0% good mood, and 2.0% poor. Caffeine consumption was moderate: 43.8% responses indicating two cups daily and 14.3% abstaining, while alcohol consumption was low, with 84.2% responses indicating no intake and only a small fraction consuming up to three servings. Among the 16 participants, 12 participants (75%) were still using a BZRA at the time of the BRAIN-e study, while 4 participants (25%) had stopped using it.

**SUPPLEMENTAL DISCUSSION**

The study population was skewed towards women (68.8%) and White individuals (87.4%). It is well established that insomnia is more prevalent in women than in men, which likely accounts for the higher proportion of women in our study. As noted in the literature, insomnia is significantly more common in women, especially due to factors such as hormonal fluctuations, pregnancy, and the greater social and caregiving roles that women often bear.^42,43^ Our study's demographic distribution is therefore consistent with these established patterns, where insomnia is more common in women. Among benzodiazepine users, particularly among older adults, prevalence of use in women was nearly twice the prevalence in men.^44,45^ Moreover, Cosci et al. found a female-to-male ratio of 64.4% to 35.6% in their study of benzodiazepine-users,^46^ which mirrors the demographic distribution in our sample. These established patterns, both in insomnia prevalence and benzodiazepine use, are consistent with the gender distribution observed in our study, where insomnia and BZRA use are more common in women. Regarding racial/ethnic differences, a national sleep survey in the United States found that insomnia diagnoses were more prevalent in White adults (10%) compared to Hispanic (7%), Asian (4%), and African American (3%) adults.^47^ Among people taking BZRAs**,** a study found that 6.5% of White were prescribed benzodiazepines, compared to 3.8% of Multiracial/Multicultural, 2.7% African American, and 2.0% Asian American.^48^ The higher proportion of White participants in our study (87.4%) is consistent with these findings; however, this overrepresentation could be attributed to challenges faced during recruitment, particularly during the COVID-19 pandemic. These challenges likely hindered participation among non-White/less educated individuals who may have had more difficulty participating in the research study during the pandemic, further exacerbating the demographic skew.

**Table S1:** Distribution of Ecological Momentary Assessments variables across all sessions

| **Variables** | | **Sessions (N=1513)** |
| --- | --- | --- |
| **Sleepiness*** | Not at all | 358 (23.7%) |
|  | Mild | 598 (39.5%) |
|  | Noticeable | 258 (17.1%) |
|  | More prominent | 299 (19.8%) |
| **Fatigue*** | Not at all | 165 (10.9%) |
|  | Mild | 765 (50.6%) |
|  | Noticeable | 342 (22.6%) |
|  | More prominent | 241 (15.9%) |
| **Cognition*** | Very good | 516 (34.1%) |
|  | Good | 671 (44.3%) |
|  | Fair | 263 (17.4%) |
|  | Poor | 63 (4.2%) |
| **Mood*** | Very good | 473 (31.3%) |
|  | Good | 681 (45.0%) |
|  | Fair | 328 (21.7%) |
|  | Poor | 31 (2.0%) |
| **Caffeine* (cup)** | 0 | 216 (14.3%) |
|  | 1 | 469 (31.0%) |
|  | 2 | 662 (43.8%) |
|  | 3 | 166 (11.0%) |
| **Alcohol*  (serving)** | 0 | 1274 (84.2%) |
|  | 1 | 142 (9.4%) |
|  | 2 | 54 (3.6%) |
|  | 3 | 43 (2.8%) |
| **BZRA  (per person)** | Yes | 12 (75%) |
|  | No | 4 (25%) |

**BZRA=benzodiazepine receptor agonist**

**Table S2.** Result of the Manual Annotation

|  | **True Speech** | **True Non-speech** |
| --- | --- | --- |
| **Predicted Speech** | 35 | 0 |
| **Predicted Non-speech** | 14 | 136 |

**SUPPLEMENTAL REFERENCES**

1. Zeng LN, Zong QQ, Yang Y, et al., *Gender Difference in the Prevalence of Insomnia: A Meta-Analysis of Observational Studies*, Front Psychiatry, 2020
2. Zhang, B., & Wing, Y. K., *Sex differences in insomnia: A meta-analysis*, Sleep, 2006
3. Reuben C, Elgaddal N, Black LI. Sleep medication use in adults aged 18 and over: United States, 2020. NCHS Data Brief, no 462. Hyattsville, MD: National Center for Health Statistics. 2023. DOI: <https://dx.doi.org/10.15620/cdc:123013>.
4. Olfson M, King M, Schoenbaum M. Benzodiazepine Use in the United States. JAMA Psychiatry.2015;72(2):136–142. doi:10.1001/jamapsychiatry.2014.1763
5. Cosci, F., Mansueto, G., Faccini, M., Casari, R., & Lugoboni, F. (2016). Socio-demographic and clinical characteristics of benzodiazepine long-term users: Results from a tertiary care center. *Comprehensive psychiatry*, *69*, 211–215. https://doi.org/10.1016/j.comppsych.2016.06.008
6. Morin, C. M., & Jarrin, D. C., *Epidemiology of Insomnia: Prevalence, Course, Risk Factors, and Public Health Burden*, Sleep Medicine Clinics, 2022
7. Race, Economic Status, and Disparities in the Receipt of Benzodiazepine Prescriptions in a Large Primary Care Sample. Dore S, Weleff J, Anand A, Thompson NR, Barnett BS. General Hospital Psychiatry. 2023 Nov-Dec;85:28-34. doi:10.1016/j.genhosppsych.2023.09.002.
